# Highly compartmentalized microbiomes in blueberry microhabitats

**DOI:** 10.3389/fmicb.2025.1732372

**Published:** 2026-01-14

**Authors:** Matteo Giese, Erika Stefani, Simone Larger, Massimo Pindo, Brian Farneti, Matteo Ajelli, Monica Cattani, Manuel Delgado-Baquerizo, Claudia Coleine, Claudio Donati

**Affiliations:** 1 University School for Advanced Studies IUSS Pavia, Pavia, Italy; 2 Unit of Computational Biology, Research and Innovation Centre, Fondazione Edmund Mach, San Michele all’Adige, Italy; 3 Unit of Fruit Crop Genetics and Breeding, Research and Innovation Centre, Fondazione Edmund Mach, San Michele all’Adige, Italy; 4 Laboratorio de Biodiversidad y Funcionamiento Ecosistémico, Instituto de Recursos Naturales y Agrobiología de Sevilla (IRNAS), CSIC, Seville, Spain; 5 Department of Ecological and Biological Sciences, University of Tuscia, Viterbo, Italy

**Keywords:** amplicon sequencing, blueberry, edaphic drivers, phyllosphere, plant microbiome, rhizosphere

## Abstract

**Introduction:**

Blueberries are considered a superfood because of their rich content of vitamins, antioxidants, and fiber, supporting multiple health benefits. Plants host complex microbiomes that play crucial roles in resistance to pathogens, productivity, and stress tolerance. Despite its importance, a comprehensive characterization of the microbiota across all major compartments of cultivated blueberry (*Vaccinium corymbosum*) is still lacking.

**Methods:**

Using high-throughput sequencing of marker genes, we provide the first integrative survey of fungal and bacterial communities associated with three distinct plant compartments: rhizosphere, leaf surface, and fruit surface, as well as the bulk soil, across 100 samples, generating datasets of over 4,000 unique fungal and 38,000 unique bacterial amplicon sequence variants (ASVs).

**Results:**

We found clear compartment differentiation, with pronounced shifts in richness, diversity, and taxonomic composition between belowground and aboveground compartments. Alpha diversity peaked in bulk soils and declined progressively toward aboveground tissues. We further detected minimal overlap across compartments, with only 9 fungal and 12 bacterial ASVs shared across all compartments. These findings challenge the soil-origin hypothesis for aboveground microbiota.

**Conclusion:**

Blueberry plants harbor highly compartmentalized microbial communities shaped by selective environmental and physiological filtering. Our findings provide a baseline for future development of targeted, compartment-specific bioinoculants aimed at enhancing beneficial microorganisms for blueberry cultivation.

## 1 Introduction

Blueberries are widely recognized as a “superfood” due to their high content of nutrients, fiber, and health-promoting bioactive and antioxidant compounds (Kalt et al., 2020). Beyond their nutritional and economic value, blueberries are a particularly suitable model for studying plant microbiome compartmentalization because they are perennial, have long-lived tissues, and show strong physiological differentiation across organs. These characteristics make them ideal for investigating how microbial communities assemble, persist, and interact across compartments over time (Wenfei et al., 2023; Jia et al., 2024; Giese et al., 2025) Throughout their entire life cycle that play crucial roles in nutrient cycling, plant health, pathogen resistance, and stress tolerance (Leach et al., 2017; Liu et al., 2020; Kandel et al., 2022). While the majority of microbial taxa are commensal, their role is often context-dependent, with taxa potentially shifting between mutualistic, commensal, and pathogenic interactions (Szymanski and Miles, 2025).

Plants are composed of various tissues, which can be typically divided into two main macro-areas—the aboveground, or phyllosphere, and belowground, or rhizosphere—that are subject to different environmental pressures (Beccari and Carmona, 2024). The phyllosphere is more exposed to climatic fluctuations (Vannette, 2020; Zhu et al., 2022), while the rhizosphere is generally more stable and resilient, showing lower temporal variability and quicker recovery after disturbance like drought (Louisson et al., 2023; Jiang et al., 2024), conversely the core microbiome of the blueberry phyllosphere has been shown to be conserved across years (Giese et al., 2025). This spatial and environmental differentiation fosters distinct microbial communities across plant tissues, contributing to the overall diversity and specialization of the plant-associated microbiome (Leveau, 2019). However, the degree of connectivity between belowground and aboveground microbiomes remains insufficiently resolved, especially in perennial plants like blueberries.

It has been suggested that soil is the major source of the taxa colonizing both the rhizosphere and phyllosphere (Xiong et al., 2021). It is still not fully addressed whether this pattern holds in long-lived plants, where microbial communities may be shaped more strongly by local adaptation than by dispersal from soil.

To better understand the factors that drive the taxonomic structure of the blueberry plants microbiome, we profiled the fungal and bacterial communities across belowground (rhizosphere and bulk soil) and aboveground (leaf surface, fruit surface) niches. Specifically, this work addresses the following research questions:

1. Do microbial communities differ consistently between above- and below-ground plant compartments?
2. To what extent do belowground microbial communities contribute to the assembly of phyllosphere microbiota, and is the rhizosphere a major source of taxa found in aerial tissues?
3. How strongly do edaphic factors (e.g., pH, salinity, ammonium) and plant genotype shape microbial diversity and community composition across the rhizosphere and associated compartments?

## 2 Materials and methods

### 2.1 Plant materials and sampling

Ten blueberry cultivars were selected from the blueberry germplasm collection of the Edmund Mach Foundation (FEM) (46.0744° N, 11.2334° E), Trentino-Alto Adige, Italy, and harvested during the 2023 growing season. To address the specific agronomic requirements of blueberry cultivation, a soil management strategy was applied to maintain the acidic pH necessary for optimal growth. For the cultivation, pellet elemental sulfur was broadcast at a rate of 100 kg/ha in November. Subsequently, a fertilization program was implemented from vegetative resumption until the beginning of harvest. Fertilization consisted of a broadcast application of a nutrient blend in the first week of each month from March to June, with the March application occasionally omitted depending on the phenological stage of the plants. The nutrient mixture was applied at a rate of 10 kg/ha (equivalent to 24 kg ammonium sulfate, 20 kg potassium sulfate, 20 kg magnesium sulfate, and 6 kg triple superphosphate per 1,000 m^2^), ensuring a balanced supply of macronutrients throughout key growth and fruiting phases.

For microbiome analysis, each cultivar was sampled in triplicate, collecting material from three individual plants. To control environmental variation, all samples were collected under stable weather conditions, even though sampling occurred on different days. Each cultivar was sampled in triplicate, with three individual plants per replicate. Statistical analyses accounted for this structure by including replicate as a random factor where appropriate. Four compartments were considered: leaves (30 samples), fruit surface (30 samples), rhizosphere (30 samples), and bulk soil (10 samples), resulting in a total of 100 samples. Fruits were harvested at stage 89 of the Biologische Bundesanstalt, Bundessortenamt und CHemische Industrie Scale. Bulk soil was collected in triplicate for each plant at approximately 1 m from the base, after removing the topsoil, and pooled to form a composite sample. Rhizosphere samples were obtained by carefully exposing and cutting roots with sterile pruning shears, minimizing damage to the plants. All collections (leaves, fruits, rhizosphere, bulk soil) were performed using sterile tweezers, trowels, and shears, and samples were immediately placed in sterile plastic bags, stored on ice in the field, and subsequently kept at −20 °C for temporary storage. Samples were kept at −20 °C for up to 2 weeks before DNA extraction. Transport from the field to the laboratory was performed on ice to maintain low temperature and minimize microbial changes.

### 2.2 Aboveground (fruit and leaf surface) DNA extraction

Samples were treated under sterile conditions. For the leaf and fruit samples, 150 mL of sterile washing solution (0.9% NaCl, 0.01% Tween 80) was added to the sample; then it was incubated on an orbital shaker at 400 rpm for 1 h (Behrens and Fischer, 2022). At the end of the incubation, the water was filtered using sterile filtration apparatus (2GPU02RE Stericup Quick Release Millipore Express PLUS 0.22 μm PES, 250 mL Merck Millipore) to recover as much biological material as possible. DNA was extracted from the filters using the DNeasy PowerWater kit QIAGEN according to the manufacturer’s protocol. A negative control was also analyzed and subjected to the same polymerase chain reaction (PCR) process as the other samples. No DNA amplification was observed in the negative control, indicating the absence of contamination during the extraction process.

### 2.3 Belowground (rhizosphere and bulk soil) DNA extraction

Using sterile forceps and spatulas, we carefully scraped off adhering soil from the roots. 250 mg of rhizosphere and bulk soil were then used for DNA extraction using the DNeasy PowerSoil Pro Kit (QIAGEN) following the manufacturer’s protocol. A negative control was included and subjected to the same PCR amplification process as the samples. No DNA amplification was observed in the negative control, confirming the absence of contamination during the extraction process.

### 2.4 Amplicon sequencing

For fungi, the ITS1 region was amplified using ITS1F (5′-CTT GGT CAT TTA GAG GAA GTAA-3′) and ITS2 (5′-GCT GCG TTC TTC ATC GAT GC-3′) primers (White et al., 1990; Gardes and Bruns, 1993) with overhang Illumina adapters. The PCR were carried out with a total volume of 25 μL, containing 1 μL of each primer (10 μM of each one), 0.25 μL of FastStart High Fidelity PCR System (Roche), 18.75 μL of nuclease-free water (Sigma–Aldrich), 2.5 [10X] Buffer, 0.5 μL dNTP and 1 μL of DNA (5–10 ng/μL). PCR were performed by using the GeneAmp PCR System 9,700 (Thermo Fisher Scientific); the conditions were: initial denaturation at 95 °C for 3 min, 35 cycles of denaturation at 95 °C for 20 s, annealing at 50 °C for 45 s, extension at 72 °C for 90 s, followed by a final extension at 72 °C for 10 min.

For bacteria, the sub-region V3–V4 of the 16S ribosomal RNA gene was amplified using the 341F (5′-CCT ACG GGN GGC WGC AG-3′) and 805R (5′-GAC TAC NVG GGT WTC TAA TCC-3′) primers (Klindworth et al., 2013) with overhang Illumina adapters. The PCR were carried out with a total volume of 25 μL, containing 5 μL of each primer (1 μM), 12.5 μL MIX KAPA HiFi HotStart ReadyMix (Roche) and 2.5 μL of DNA (5–10 ng/μL).

PCR were performed by using the GeneAmp PCR System 9700 (Thermo Fisher Scientific); the conditions were: initial denaturation at 95 °C for 5 min, 35 cycles of denaturation at 95 °C for 30 s, annealing at 55 °C for 30 s, extension at 72 °C for 30 s, followed by a final extension at 72 °C for 5 min.

The obtained amplicons were purified using the CleanNGS (CleanNA), following the manufacturer’s instructions. Afterward, a second PCR was used to apply dual indices and Illumina sequencing adapters Nextera XT Index Primer (Illumina), by 7 cycles PCR (16S Sequencing Library Preparation, Illumina). The amplicon libraries were purified using CleanNGS (CleanNA), and the quality control was performed on a Tapestation 4150 platform (Agilent Technologies, Santa Clara, CA, United States). Finally, all barcoded libraries were pooled in an equimolar way and sequenced on an Illumina^®^ MiSeq (PE300) platform in pair ends (2 × 300 bp) (MiSeq Control Software 2.5.0.5 and Real-Time Analysis software 1.18.54.0). Library construction and Next-Generation Sequencing were performed at the sequencing platform at FEM. Negative controls were also sequenced to further exclude potential contaminants.

### 2.5 Chemical physical analysis

Physicochemical characterization of soil samples included texture, pH, salinity, contents of total NH₄^+^ and phosphorus (P). These characterizations were performed following standard methods (Siles et al., 2023).

### 2.6 Bioinformatic analysis

The demultiplexed raw reads were analyzed using the MICCA (MICrobial Community Analysis) v1.7.2 bioinformatics pipeline (Albanese et al., 2015). Sequence pairs were merged to obtain consensus sequences. Primers were trimmed and sequences that did not contain these primers were discarded. Quality filtering was performed using an expected error rate of 0.75 for ITS and 16S, with the minimum read length set to 200 for ITS and 400 for 16S. Reads were clustered into amplicon sequence variants (ASVs) using the UNOISE protocol (Edgar, 2016). A total of 38,259 ASVs for bacteria and 4,658 ASVs for fungi were found. Finally, taxonomy was assigned using AMPtk (Amplicon ToolKit) v1.5.1 pipeline (Palmer et al., 2018) with the database UNITE v10.3 (Abarenkov et al., 2024) for the fungal component and Rdp_16s_v16 (Edgar, 2018; Wang and Cole, 2024) for the bacterial component. To ensure the absence of contaminants after sequencing, the R package “Decontam” version 1.26.0 (Davis et al., 2018) was used, with negative controls as reference. The analysis confirmed the absence of contamination in the dataset. To reduce the influence of extremely rare taxa and potential sequencing artifacts, we applied a prevalence filter prior to all compartment overlap analyses. Specifically, only ASVs present in at least 5% of samples within a given compartment were retained. This step ensured that downstream comparisons reflected biologically meaningful microbial taxa rather than low-abundance noise.

### 2.7 Statistical analysis

The ITS dataset included 4,289 unique ASVs, with a total of 605,540 raw, non-rarefied reads, which decreased to 501,813 reads after rarefaction. The 16S dataset comprised 38,152 unique ASVs, with 4,959,760 raw, non-rarefied reads, reduced to 3,204,000 reads after rarefaction. The number of raw reads for each sample is reported in Supplementary Table 1. Rarefaction was applied to standardize sequencing depth across samples, which enabled reliable comparisons of microbial and fungal community diversity. The rarefaction depth was chosen based on a trade-off between retaining samples and capturing true diversity, which balanced coverage and diversity loss. Samples from all compartments were rarefied to 9,000 reads for bacteria and 10,000 for fungi. Two alpha diversity indices were computed: Chao1 and Shannon diversity (Kim et al., 2017; Willis, 2019; Finn, 2024), using the “estimate_richness” function from the R package Phyloseq v1.46.0 (McMurdie and Holmes, 2013). Chao1 and the Shannon indices were used to estimate richness and diversity, respectively. The Wilcoxon-Mann–Whitney test was used to assess significance of difference in alpha diversity among groups with Holm–Bonferroni correction for multiple testing. For beta diversity analysis, the Bray–Curtis dissimilarity matrix was used to assess the difference between the plant tissues, which were visualized with a principal coordinate analysis (PCoA). Statistical significance was assessed using permutational multivariate analysis of variance (PERMANOVA) with pairwiseAdonis v0.4 R package (Martinez-Villegas et al., 2024). The parameters considered in this analysis were difference in tissues, and difference in plant cultivar compared to the reference set of pairwise distances between samples from the same plant cultivar and tissues.

For differential abundance analysis and Spearman’s correlations between alpha diversity and edaphic factors, the Microeco v1.13.0 R package (Liu et al., 2021) was used, applying Random Forest classification along with a zero-inflated log-normal (ZILN) model-based differential test, particularly suitable for identifying differentially abundant taxa in sparse datasets (Paulson et al., 2013). Tissue overlap was calculated by converting the ASVs table into a presence/absence matrix, where ASVs were considered present if their abundance was greater than zero. ASVs were defined as present in a tissue if they were detected in at least one sample and present in at least 9 out of 10 cultivars. For visualization, the UpSetR v1.4.0 package was used (Conway et al., 2017). Distance-based redundancy analysis (dbRDA) was performed with Microeco v1.13.0 R package; this constrained ordination method related community dissimilarity matrices to environmental variables, enabled the identification of factors shaping community composition (Yao et al., 2023), moreover we employed envfit correlation from “vegan” to fit the environmental variables onto the PcOA ordination (Kuusisto et al., 2025). The environmental variables used in envfit were NH₄^+^, P, pH, salinity and texture.

## 3 Results

### 3.1 Niche partitioning shapes the microbiome of blueberries

**Figure 1:**
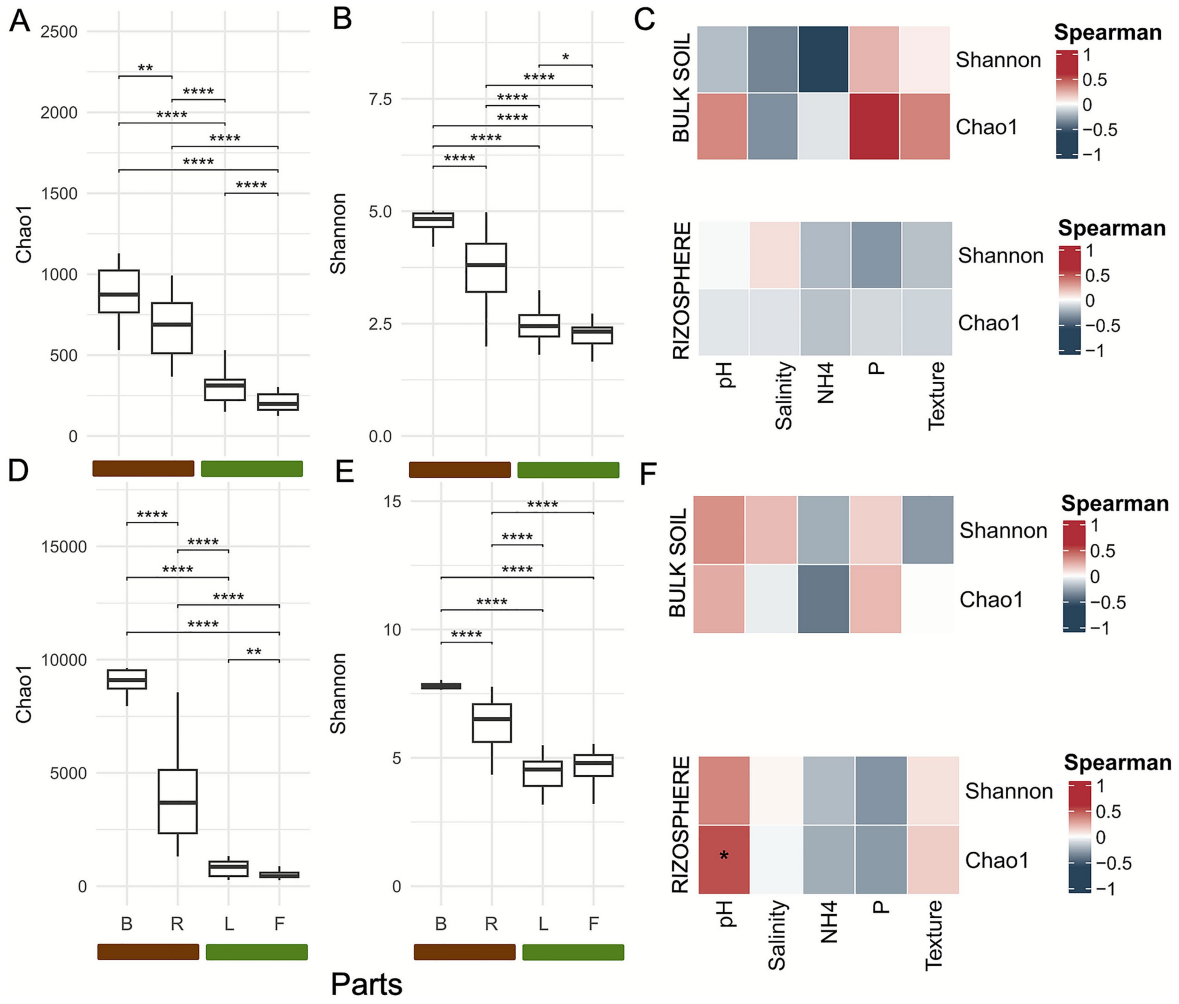
Alpha diversity shows significant differences across compartments. **(A)** Alpha diversity Chao1 index of fungal communities in bulk soil, fruit and leaf epiphyte tissues, and rhizosphere. **(B)** Alpha diversity Shannon index of fungal communities in bulk soil, fruit and leaf epiphyte tissues, and rhizosphere. **(C)** Influence of physicochemical parameter on fungi alpha indices of bulk soil and rhizosphere. **(D)** Alpha diversity Chao1 index of bacterial communities in bulk soil, fruit epiphyte, leaf epiphyte tissues, and rhizosphere. **(E)** Alpha diversity Shannon index of bacterial communities in bulk soil, and rhizosphere. **(F)** Influence of physicochemical parameter on bacteria alpha indices of bulk soil and rhizosphere (*p*-value: “****” = 0.0001; “***” = 0.001; “**” = 0.01; “*” = 0.05). B: bulk soil; F: fruit epiphytes; L: leaf epiphytes; R: rhizosphere of blueberry cultivars.

We first explored microbial alpha and beta diversity to assess how communities differ in richness across plant compartments. After rarefaction, we computed the Chao1 estimator of species richness and the Shannon entropy index to characterize microbial richness and diversity in each compartment. Richness was highest in bulk soil and the rhizosphere both for fungi (Figure 1A) and bacteria (Figure 1D), with a marked decline on the leaf and fruit surfaces. Shannon diversity index, both for fungi (Figure 1B) and bacteria (Figure 1E), shows that bulk soil and rhizosphere exhibit significantly higher diversity compared to the leaf and fruit epiphytes (Supplementary Table 2).

To explore how soil properties influence belowground microbial communities, we measured soil physico-chemical parameters (Supplementary Figure 1 and Supplementary Table 3). Most variables differed significantly except texture. Pairwise correlations revealed a negative correlation between pH and both salinity and ammonium, and a positive correlation between texture and phosphorus (Supplementary Figure 3). The influence of environmental factors on alpha diversity was assessed using Spearman’s correlation analysis (Figures 1C–F). The only significant correlation was observed in the bacterial community of the rhizosphere, where Chao1 was positively correlated with pH (*ρ* = 0.517, *p* = 0.034), whereas no significant correlations were found for Shannon. In bulk soil no statistically significant correlations were detected after *p*-value adjustment, although some weak to moderate non-significant trends were present.

**Figure 2:**
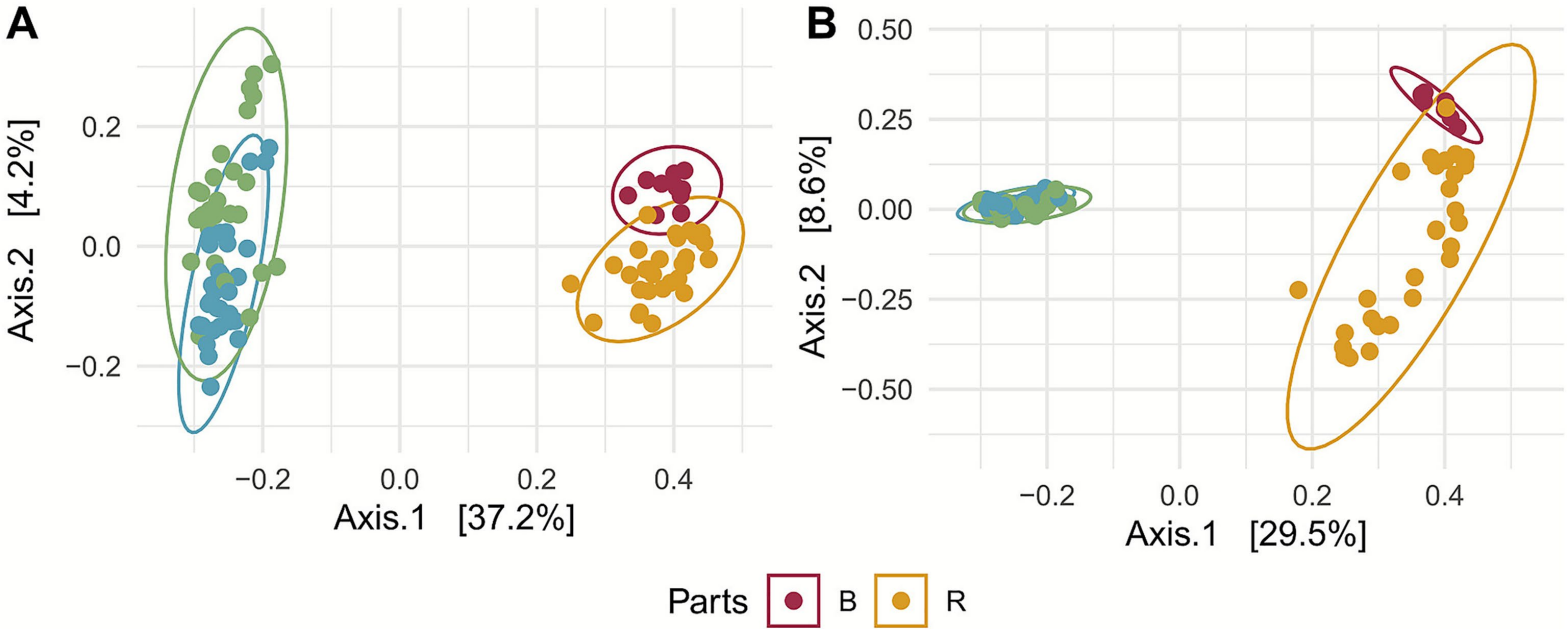
Beta diversity based on UniFrac distances showing significant differences across tissues. **(A)** PCoA plot of fungal communities, illustrating clear separation among bulk soil (B), rhizosphere (R), fruit epiphytes (F), and leaf epiphytes (L). The first two axes explain the largest proportion of variance in community dissimilarity, highlighting strong compartment-driven structuring of fungal assemblages. **(B)** PCoA plot of bacterial communities, likewise showing distinct clustering by tissue type, with belowground (B, R) communities clearly separated from aboveground (F, L) epiphytic communities. The spread of points reflects differences in community turnover, indicating that environmental and physiological gradients strongly shape bacterial beta diversity across compartments.

PCoA of beta diversity revealed two main clusters separating aboveground from belowground communities for both fungi (Figure 2A) and bacteria (Figure 2B). PERMANOVA also indicated significant differences among aboveground and belowground parts of the plants, both for fungi (*p*-value = 0.001, *R*^2^ = 0.3963) and bacteria (*p*-value = 0.001, *R*^2^ = 0.3352). Additionally, pairwise PERMANOVA confirmed that microbial communities differed significantly between plant compartments. For both fungi and bacteria, aboveground compartments (fruit and leaf) were distinct from belowground compartments (bulk soil and rhizosphere), whereas differences between bulk soil and rhizosphere were smaller (Supplementary Table 5), highlighting the close connection between rhizosphere and surrounding soil.

**Figure 3:**
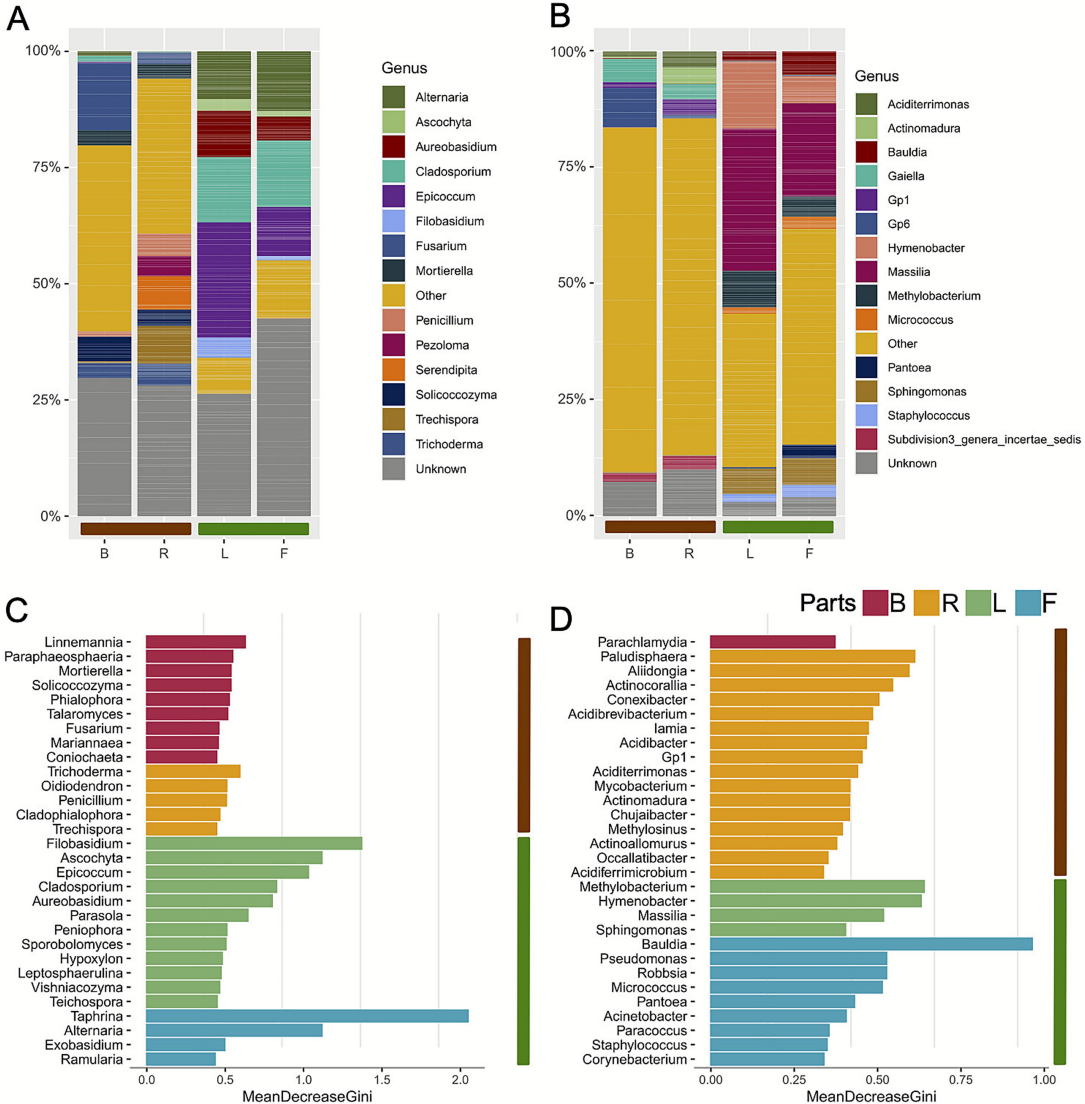
Composition of the 10 most abundant taxa for fungal and bacterial communities in different plant compartments of blueberry cultivars. **(A)** Stacked bar charts showing the relative abundance of fungal genera in the four compartments. **(B)** Stacked bar charts showing the relative abundance of bacterial genera in the four compartments. **(C)** Fungal keystone taxa (at the genus level) for compartment: bulk soil (dark violet), fruit epiphytes (sky blue), leaf epiphytes (green) and rhizosphere (yellow). **(D)** Bacterial keystone taxa for compartment bulk soil (dark violet), fruit epiphytes (sky blue), leaf epiphytes (green) and rhizosphere (yellow). B: bulk soil; F: fruit epiphytes; L: leaf epiphytes; R: rhizosphere.

We then investigated the relative abundance of the main microbial groups. We identified a total of 9 fungal phyla (Supplementary Table 6) and 10 bacterial phyla (Supplementary Table 7), together with 14 genera for both fungi and bacteria (Figures 3A,B). At the phylum level, fungi (Supplementary Figure 2A) showed a dominance of *Ascomycota* across all compartments except for the rhizosphere. Following in decreasing order, we identified *Basidiomycota*, *Mortierellomycota*, and *Rozellomycota*. For bacteria (Supplementary Figure 2B), *Proteobacteria* emerged as the dominant phylum, followed by *Actinobacteria* in all the compartments; whereas both the rhizosphere and bulk soil displayed a higher relative abundance of *Acidobacteria*. This pattern already highlights a compositional difference among the different plant-associated compartments. At the genus level (Supplementary Table 7), compositional differences became even more pronounced for fungi such as *Mortierella*, which was particularly abundant belowground; *Serendipita* and *Pezoloma*, typical of the rhizosphere; whereas *Ascochyta* and *Epicoccum* were characteristic of aboveground compartments, revealing a clearly different community structure. A similar pattern was observed for bacteria (Supplementary Table 8): genera such as *Aciditerrimonas* and *Actinomadura* were more prevalent in the belowground compartments, whereas *Massilia* and *Hymenobacter* were more abundant in the aboveground compartment. Notably, a significant proportion of the fungal community, exceeding 25%, remained unclassified across all plant tissues analyzed. This result contrasts sharply with observations in bacterial communities, where the abundance of unclassified taxa was considerably lower. At the genus level, bacterial communities showed a greater degree of diversity, with the “Other” category which represents the most abundant group across all compartments and including all taxa not within the top 15 most abundant.

### 3.2 Distinct predictive biomarkers taxa reveal niche differentiation across plant compartments

To more precisely define the taxa specifically associated with each compartment, or predictive biomarkers taxa, we trained a random forest model that predicts the compartment using taxonomic profiles at the genus level as input (Figures 3C,D). We identified several predictive biomarkers taxa that exhibit compartment-specific associations. For fungi, the bulk soil was characterized by the presence of *Linneamannia*, *Talaromyces* and *Mortierella*, while we found in the rhizosphere genera such as *Trichoderma* and *Sporothrix* as predictive biomarkers taxa. The leaf surface exhibited a distinct set of predictive biomarkers taxa, including *Epicoccum*, *Ascochyta*, *Cladosporium*, *Aureobasidium*, and *Peniophora* as well as *Vishniacozyma*. In contrast, the fruit epiphytic community was enriched in *Taphrina*, *Alternaria* and *Curvibasidium*. Similarly, bacterial predictive biomarkers taxa varied across compartments. In bulk soil, we identified *Parachlamydia* while the rhizosphere harbored *Aciditerrimonas*, *Acidibrevibacterium*, *Acidibacter*, *Mycobacterium*, *Iamia*. The leaf surface was dominated by *Methylobacterium*, *Sphingomonas* and *Massilia*. The fruit surface microbiota was enriched in *Pseudomonas*, alongside *Pantoea*, *Micrococcus*, and *Acinetobacter*.

### 3.3 Environmental parameters exerts a stronger influence on bacterial than fungal communities in soil

**Figure 4:**
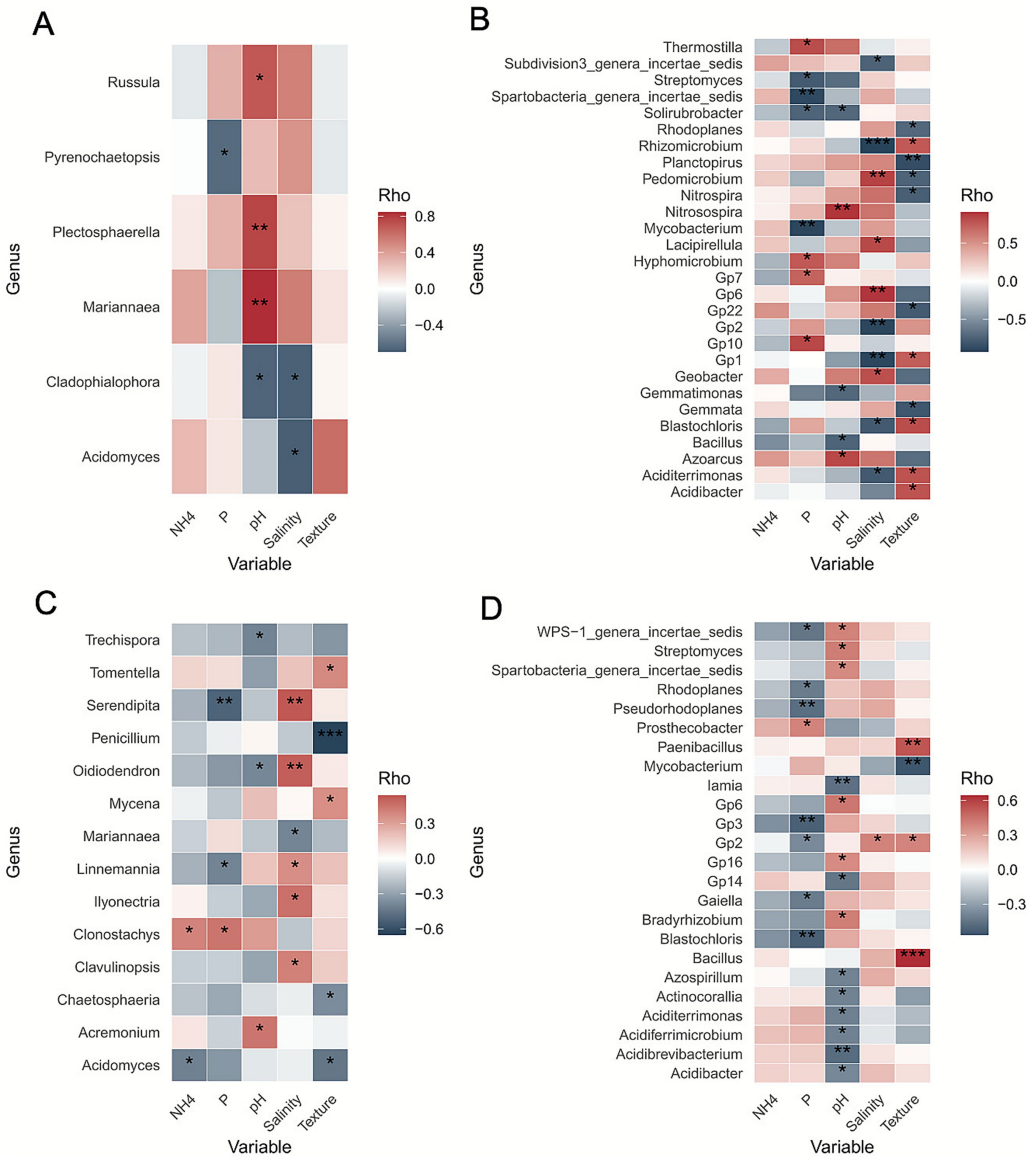
Spearman’s correlation (*ρ*) between microbial genera and soil physicochemical parameters across belowground compartments. **(A)** Correlation between fungal genera and soil factors in bulk soil, highlighting genera whose abundance is positively or negatively associated with pH, salinity, ammonium, phosphate, and soil texture. **(B)** Correlation between bacterial genera and soil factors in bulk soil, showing key taxa influenced by physicochemical gradients. **(C)** Correlation between fungal genera and soil parameters in the rhizosphere, illustrating how nutrient availability and other edaphic factors shape community composition. **(D)** Correlation between bacterial genera and soil parameters in the rhizosphere, revealing taxa strongly associated with specific soil properties. (*p*-value: “****” = 0.0001; “***” = 0.001; “**” = 0.01; “*” = 0.05).

To assess the influence of edaphic factors on microbial taxa across different soil niches we analyzed Spearman’s correlation coefficients (*ρ*) between microbial genera (Supplementary Tables 10, 11) (in both rhizosphere and bulk soil) and physico-chemical parameters (NH₄^+^, P, pH, salinity and texture). We found that pH was the primary influencer of the abundance of fungal genera (Figure 4A). For some genera relative abundance increased with pH (as for example *Plectosphaerella*) while in other cases (e.g., *Cladophialophora*) the correlation was negative. Additionally, salinity and phosphorus were identified as influential factors, both showing negative correlations (respectively with *Acidomyces* and *Pyrenochaetopsis*), whereas different concentrations of NH₄^+^ and texture did not show any effect. In the rhizosphere, several fungal taxa were notably influenced by the edaphic factors analyzed (Figure 4C). For example, in the rhizosphere *Oidiodendron* and *Serendipita* were influenced by P concentration, pH and salinity; specifically, *Oidiodendron* showed a negative correlation with pH and a positive correlation with salinity, while *Serendipita* was negatively correlated with P and positively correlated with salinity. Interestingly, we found some genera that were influenced by different factors depending on the compartment analyzed. For example *Acidomyces* was negatively correlated with salinity in bulk soil, while in the rhizosphere was negatively correlated with NH₄^+^ and texture. The correlation with salinity was not significant.

Regarding bacteria, they were primarily influenced by salinity and soil texture, with additional contribution from pH and phosphorus levels (Figure 4B), while NH₄^+^ did not exhibit any significant effect. In the rhizosphere, pH emerged as the most influential factor shaping bacterial community composition, showing both positive and negative correlations with different taxa. Phosphorus and soil texture also influenced the taxonomic composition, while salinity appeared to have a relatively minor effect. As observed in bulk soil, NH₄^+^ showed no significant influence on bacterial taxa in the rhizosphere (Figure 4D). In the rhizosphere, *Gp 2* was correlated negatively with phosphorus concentration while it was positively correlated with both salinity and texture. *Iaima* showed a negative correlation with pH. Also for bacteria we found that some taxa, such as *Acidibacter*, responded differently depending on the compartment analyzed. In particular, in the rhizosphere *Acidibacter* was negatively correlated only with pH while in bulk soil was positively correlated only with soil texture.

### 3.4 Soil physico-chemical factors drive microbial community structure in rhizosphere and bulk soil

**Figure 5:**
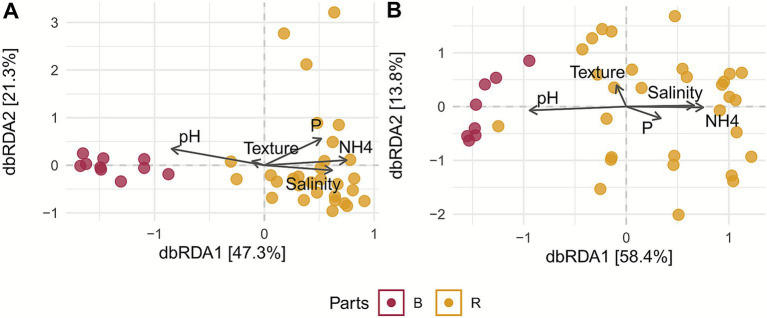
dbRDA based on UniFrac distances showing significant differences across tissues. Vector directions and lengths indicate the magnitude and orientation of environmental gradients, with pH, salinity, ammonium, and phosphate representing the strongest predictors of belowground microbial structure. The proportion of variance explained by the constrained axes is reported on each plot, highlighting the overall influence of physicochemical variables on community assembly. **(A)** dbRDA illustrates how soil physicochemical factors shape fungal community composition in bulk soil and rhizosphere. **(B)** dbRDA illustrates how soil physicochemical factors drive bacterial community composition in bulk soil and rhizosphere. B: bulk soil; F: fruit epiphytes; L: leaf epiphytes; R: rhizosphere of blueberry cultivars.

The distance-based redundancy analysis (dbRDA) of beta diversity revealed distinct differences in fungal community composition between rhizosphere and bulk soil samples and how the soil physico-chemical parameters significantly explained the variation in microbial community structure (Figures 5A,B). Both for bacteria and fungi dbRDA analyses showed a clear separation between rhizosphere (R) and bulk soil (B) samples, accounting for 47.3% of the variance in fungal communities and 58.4% in bacterial communities. This separation was primarily driven by edaphic factors such as salinity, ammonium (NH₄^+^) concentration, and pH. Salinity and NH₄^+^ showed a positive association with rhizosphere samples, whereas pH was negatively correlated with them. The regression of environmental variables with ordination axes (function “envfit,” See methods) revealed significant associations between environmental variables and microbial community structure in the dbRDA ordination space (Supplementary Table 11). For fungal communities, pH, available phosphorus, ammonium (NH₄^+^), and salinity were significantly correlated with community composition, while soil texture was not significant. pH aligned primarily with dbRDA1, while phosphorus was oriented along dbRDA2, indicating distinct environmental gradients influencing fungal distribution. For bacterial communities, pH and ammonium were the strongest predictors. Salinity and phosphorus were also significant but explained less variance. Moreover, we assessed the influence of plant genotype on microbial community composition, which revealed significant differences for both fungi and bacteria. Genotype explained 42.6% of the variance in bacterial communities and 40.4% of the variance in fungal communities (Supplementary Table 13).

### 3.5 Sharp microbial partitioning between above- and belowground compartments

**Figure 6:**
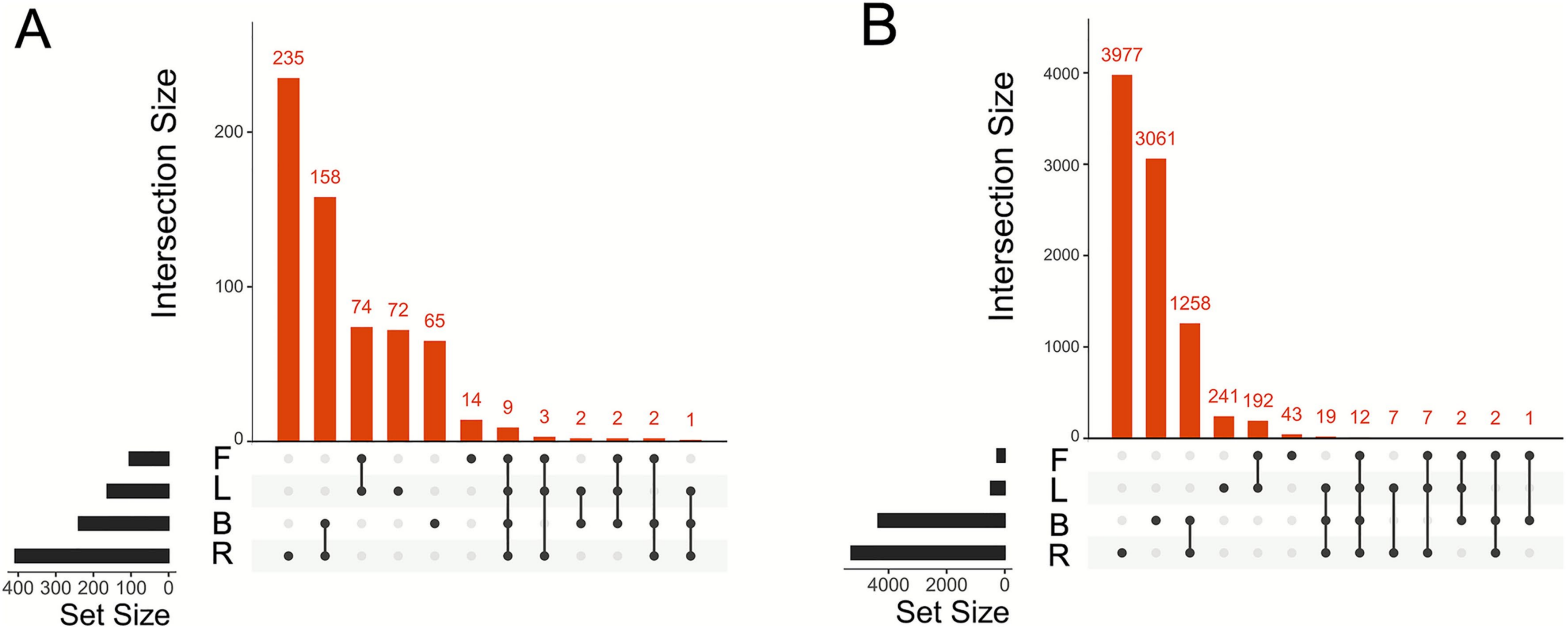
Upset plot of overlap of different compartments; it was calculated with presence/absence matrix, where ASVs were considered present if their abundance was greater than zero. ASVs were defined as present in a tissue if they were detected in at least one sample and occurred in at least 9 out of 10 blueberry cultivars. **(A)** Upset plot of fungi at ASVs level. **(B)** Upset plot of bacteria at ASVs level. (B: bulk soil; F: fruit epiphytes; L: leaf epiphytes; R: rhizosphere).

After identifying the different predictive biomarkers taxa, we further analyzed the distribution of ASVs to measure the overlap between aboveground and belowground compartments, for both bacterial and fungal communities (Figures 6A,B) in order to assess whether there is a flow of taxa between the belowground and aboveground compartments. We found only 9 ASVs and 12 ASVs shared among all compartments for fungi and bacteria, respectively. In the belowground compartments, we identified 235 ASVs exclusive to the fungal rhizosphere and 3,977 ASVs for bacteria, 65 ASVs exclusive to fungal bulk soil and 3,061 ASVs for bacteria, and 158 fungal ASVs and 1,258 bacterial ASVs shared between rhizosphere and bulk soil. In contrast, in the aboveground compartments, 72 fungal ASVs and 241 bacterial ASVs were unique to the leaf surface, 14 fungal and 43 bacterial ASVs were exclusive to the fruit surface, and 74 fungal ASVs and 192 bacterial ASVs were shared between these two compartments.

To better understand the distribution of ASVs shared across all compartments (9 for fungi and 12 for bacteria), we analyzed their relative abundances for fungi (Supplementary Table 14) and bacteria (Supplementary Table 15). Fungal ASVs (Supplementary Figure 4A) exhibited distinct abundance patterns between aboveground (green) and belowground (brown) compartments. Typically, ASVs showed asymmetrical abundance distributions. For instance, *Penicillium* was notably enriched in the rhizosphere but less abundant aboveground. In contrast, *Fusarium* predominated in bulk soil, showing reduced abundance aboveground. Conversely, genera such as *Epicoccum* and *Cladosporium* consistently showed higher abundance in aboveground compartments. A similar trend was observed for bacterial communities (Supplementary Figure 4B). For instance, *Methylobacterium* and *Sphingomonas* were more abundant in the epiphytic communities of aboveground compartments, whereas *Bacillus* and *Microvirga* exhibited higher relative abundance in belowground habitats and were relatively scarce in the phyllosphere.

## 4 Discussion

Despite their increasing economic importance, we know relatively little about the microbiome of blueberry, a woody perennial plant widely cultivated worldwide. While previous studies have investigated this species (Song et al., 2021; Dong et al., 2022; Li et al., 2024; Giese et al., 2025; Szymanski and Miles, 2025), our study provides one of the first simultaneous characterizations of blueberry microbiota across leaf and berry surfaces, rhizosphere, and neighboring bulk soil. We show a clear and strong differentiation between aboveground and belowground microbiomes, reflecting compartment-specific environmental conditions, extending findings from previous research (Song et al., 2021; Dong et al., 2022; Li et al., 2024; Giese et al., 2025; Szymanski and Miles, 2025). Key edaphic factors (pH, salinity, ammonium) strongly influenced belowground microbial diversity, highlighting potential levers for microbiome management in blueberry cultivation. While our results derive from one site, the patterns observed likely reflect general compartmental trends in blueberry microbiomes.

The alpha diversity analysis across plant compartments revealed marked and significant differences in biodiversity, with higher richness and diversity in bulk soil and rhizosphere compared to aboveground compartments (bulk soil → rhizosphere → leaf epiphytes → fruit epiphytes). This trend may be attributed to the strong selective pressures imposed by the limited availability of resources and the inherent challenges of colonizing the leaf and fruit surface (Müller et al., 2016; Pawlowska, 2024; Wang and Kuzyakov, 2024). While some studies reported contrasting results (Yang et al., 2022; Faticov et al., 2023), a study on *Pinus koraiensis* (Deng et al., 2022; Peng et al., 2025) clearly demonstrated the differentiation of alpha diversity between phyllosphere, rhizosphere, and bulk soil. Moreover we found that edaphic factors can influence bacterial alpha diversity in the soil, consistently with previous studies (Wang et al., 2019; Tan et al., 2020).

Beta diversity analysis confirms a strong habitat segregation among below- and aboveground. In particular, the rhizosphere, enriched by root exudates containing organic compounds and nutrients, promotes the selective proliferation of specific microbial taxa, resulting in higher microbial abundance (Coleman-Derr et al., 2016; Bao et al., 2020; Deng et al., 2022). Conversely, the phyllosphere—characterized by lower environmental stability and reduced organic matter—harbors less diverse bacterial communities, which are often more susceptible to environmental fluctuations (Vacher et al., 2016; Cavicchioli et al., 2019; Bao et al., 2020). These results reflect the profound physiological and functional differences between the two habitats (Zhou et al., 2019; Genitsaris et al., 2020). A similar trend has also been observed for fungal communities, as reported in other studies on *Bothriochloa ischaemum* and *Broussonetia papyrifera* (Chen et al., 2020; Jia et al., 2020). Multiple regression of environmental variables show that edaphic factors, particularly pH and phosphorus, were important drivers of fungal community composition, accounting for more than 72% of the variance. This result is consistent with other studies (Sun et al., 2022; Yang et al., 2024). Ammonium and salinity also had significant, albeit smaller, effects, indicating the importance of nutrient availability and osmotic stress in fungal distribution (Shi et al., 2021). In contrast, soil texture exhibited no significant association, which means that chemical factors, rather than physical soil structure were the major ecological filters in this system. Similar trends were observed in bacterial communities, where pH and ammonium were the primary determinants of community assembly (Sun et al., 2022; Cao et al., 2024).

Specifically, our results revealed clear differences between belowground (rhizosphere and bulk soil) and aboveground (leaf and fruit) microbial communities, highlighting strong compartmentalization and niche-specific microbial assemblages. This was seen in other plants such as grapevine (Berlanas et al., 2019; Bill et al., 2021; Patanita et al., 2022).

The bacterial community composition varies across different plant compartments, reflecting compartment-specific taxonomic differentiation. Relative abundance analysis revealed that at phylum level *Proteobacteria* was the dominant taxa and the second most abundant taxa was *Actinobacteria*, in line with other studies on blueberries (Wang et al., 2022). *Acidobacteria* were highly abundant in both the rhizosphere and bulk soil, as shown in (Kalam et al., 2020; Jia et al., 2024), likely reflecting the plant’s preference for acidic soil conditions (Harmer, 1945; Ochmian et al., 2020). Beyond relative abundance, it is equally important to identify predictive biomarkers, microbial groups that, even if not very abundant, strongly influence community structure and ecosystem functions (Banerjee et al., 2018). In the epiphytic fungal communities of fruits, *Taphrina* and *Alternaria* emerged as the main predictive biomarkers. *Alternaria* is a genus frequently found in fruit environments, such as apples, while *Taphrina* is known as a pathogen associated with plant tissues, as also reported in recent studies on *Arabidopsis thaliana* (Christita et al., 2021). Moreover, we detected the presence of *Ascochyta*, a genus responsible for some of the most significant diseases affecting legumes (Bretag et al., 2006; Jha et al., 2022; Singh et al., 2022)., whose presence in blueberries has not been previously reported or discussed, despite being documented in association with other plant species (Chen et al., 2025). Leaves host a distinct set of predictive biomarkers taxa, including genera such as *Cladosporium*, a well known pathogen of maize and tomato (Iida et al., 2018; Qi et al., 2023), *Filobasidium* and *Aureobasidium*. The latter is known for its antifungal properties and ability to promote plant growth (Zexuan et al., 2025) and for these reasons is employed in agronomic applications both for biocontrol and productivity (Pavicich et al., 2022). Although each plant tissue hosts distinct fungal communities, leaves and fruits appear particularly selective. Microorganisms in these aboveground habitats face variable stresses—UV radiation, temperature and humidity changes, and limited nutrients—which likely favor fungi with specialized adaptations (Beattie and Lindow, 2007; Vorholt, 2012). This could explain the prevalence of taxa with either pathogenic capabilities, such as Cladosporium and Taphrina, or protective/beneficial functions, such as Aureobasidium, which can provide both antifungal activity and plant growth promotion (Zexuan et al., 2025). In bulk soil, predictive biomarkers taxa included saprophytic fungi such as *Mortierella*, *Linnemannia*, and *Talaromyces*. Saprotroph taxa play key roles in biogeochemical cycles and in the mineralization of organic matter (Crowther et al., 2012; Di Francesco et al., 2023). We also find *Fusarium,* which is a widely distributed genus known for its pathogenicity and for producing mycotoxins in cereals (Goncharov et al., 2020). In the rhizosphere predictive biomarkers taxa included the genus *Trichoderma,* which is known for his plant-beneficial functions (e.g., antagonism of pathogens, stimulation of root growth) and contributions to plant defense by producing volatile compounds and effector proteins in concert with plant growth-promoting bacteria (Xu et al., 2021; Bouwman et al., 2025).

Regarding bacterial communities, fruits exhibit a distinct microbial composition, notably characterized by the presence of *Pseudomonas*, a well-known pathogen of various fruit crops (Banoo et al., 2020; Warring et al., 2024). Genomic analyses of epiphytic *Pseudomonas* (e.g., *Pseudomonas* sp. 14A) show metabolic adaptations to epiphytic lifestyles, enabling efficient colonization and nutrient exploitation within these microhabitats (Medina-Salazar et al., 2022). Additionally, we found taxa from the genus *Robbsia,* which has been detected in various fruits and considered to have a potential phytopathogenic role (Lopes-Santos et al., 2017). Leaves show a predominance of *Methylobacterium*, known for promoting growth through plant hormone production, together with *Sphingomonas* and *Massilia*, which are frequently isolated from leaf surfaces (Vorholt, 2012; Jackson et al., 2013). *Sphingomonas*, in particular, is associated with the production of plant growth-stimulating factors (Lombardino et al., 2022), while *Methylobacterium* produce UVA-absorbing compounds to mitigate UV damage and possess specialized metabolism for utilizing compounds like methanol released from plant cell walls (Lazazzara et al., 2021; Guzmán-Guzmán et al., 2024). The rhizosphere is dominated by *Actinobacteria* such as *Actinomadura*, known for producing volatile antimicrobial compounds (e.g., hydrogen cyanide) which contribute to pathogen biocontrol and promotes plant health (Boukhatem et al., 2022). *Mycobacterium*, frequently detected in the rhizosphere of crops like tomato (Bouam et al., 2018), is associated with the degradation of complex organic compounds (Walsh et al., 2019). In addition to these compartment-specific environmental drivers, plant genotype also significantly influenced rhizobial microbial community composition, explaining over 40% of the variance for both bacterial and fungal communities. This suggests that host genetics, together with local environmental conditions, jointly shape the assembly of plant-associated microbiomes (Darriaut et al., 2022). This knowledge has the potential to inform the design of targeted bioinoculants for leaves, fruits, or the rhizosphere, aimed at enhancing plant resistance to pathogens or improving nutrient uptake efficiency (Müller et al., 2016).

Previous studies have reported significant microbiome overlap between plant compartments, suggesting microbial continuity between above- and below-ground tissues and implying colonization via environmental vectors or systemic mechanisms (Coleman-Derr et al., 2016; Bao et al., 2020; Xu et al., 2022). In a study in grapevine, the derivation of the phyllosphere microbiota from soil microbiota was proposed (Zarraonaindia et al., 2015). In contrast, our data indicate strong microbiome compartmentalization. We found very limited ASV sharing across all compartments. This suggests limited microbial translocation between belowground and aboveground compartments and indicates that the microbial communities associated with leaves and fruits are unlikely to originate from the rhizosphere or bulk soil. Focusing on the small number of ASVs shared across all compartments, we found even for these ubiquitous taxa there was a strong preference for one of the two habitats. While certain genera were predominantly abundant in belowground habitats, others were more enriched in aboveground tissues. For instance, *Penicillium* was abundant in the rhizosphere, *Fusarium* in bulk soil, while *Epicoccum* and *Cladosporium* were abundant in aerial surfaces. These findings further support the concept of “compartmentalization,” where each plant compartment selects specific microbial communities shaped by local pressures (Ramakrishnan et al., 2024).

Spearman’s correlation coefficients (*ρ*) between microbial genera and soil parameters suggest that both soil fungal and bacterial community structures are shaped by a combination of soil nutrient availability, pH, salinity, and texture. However, bacteria appeared more responsive to phosphorus and pH, contrary to what has been reported in another study (Zhi et al., 2025), while fungal communities showed less pronounced responses overall, and a specific sensitivity to NH₄^+^ that was not detected in bacteria.

This study provides the first full-scale characterization of the microbiome across all major compartments of cultivated blueberry. We reveal strong microbial compartmentalization, with distinct fungal and bacterial communities shaped by local environmental and physiological factors. The rhizosphere hosted the highest diversity, while aboveground tissues harbored less diverse but more specialized microbiota. The limited taxonomic overlap observed between rhizosphere and phyllosphere compartments suggests that soil may not be the major source of the aerial microbiota and highlights the potential role of compartment-specific filtering mechanisms. However, these patterns are based on ASV-level data, and further work using strain-resolved sequencing and assessment of aerial dispersal sources (e.g., air, insects, irrigation) is needed to confirm microbial origin and transmission dynamics. Edaphic factors such as salinity, pH, ammonium, and phosphate emerged as key drivers of diversity. These findings offer a foundation for developing targeted microbial inoculants and advancing our understanding of plant microbiome assembly.

Our study also presents limitations. First, all samples were collected from a single agricultural site, which introduces potential site-specific environmental bias and limits the generalizability of our findings across different climates, soil types, and management systems. Because all cultivars experienced the same local conditions, genotype-environment interactions could not be disentangled, and some of the observed patterns may reflect environmental uniformity rather than species-specific responses. Second, we lacked detailed environmental metadata for aboveground plant compartments. Key variables such as canopy microclimate, humidity, UV exposure, irrigation regime, and possible pesticide or fungicide applications were not recorded. These unmeasured factors are known to strongly influence phyllosphere community assembly and may have contributed to the pronounced separation observed between above- and below-ground microbiomes. Moreover, given the limited number of replicates per cultivar (*n* = 3), genotype effects could still be partially influenced by plant-specific factors such as root structure or age. Future studies incorporating multi-site sampling, comprehensive environmental monitoring, and an explicit evaluation of microbial dispersal mechanisms will be essential to validate and extend the ecological patterns identified here.

## Data Availability

The data presented in this study are publicly available. The data can be found here: https://www.ebi.ac.uk/ena, accession PRJEB98254 and PRJEB82098.
