# Supplementary material for: Highly compartmentalized microbiomes in blueberry microhabitats
Source: Front Microbiol. 2026 Jan 14;16:1732372. doi: 10.3389/fmicb.2025.1732372 (PMC12847440; doi:10.3389/fmicb.2025.1732372)
Supplement: Supplementary file 1 [file Supplementary_file_1.docx]

**Supplementary figure**


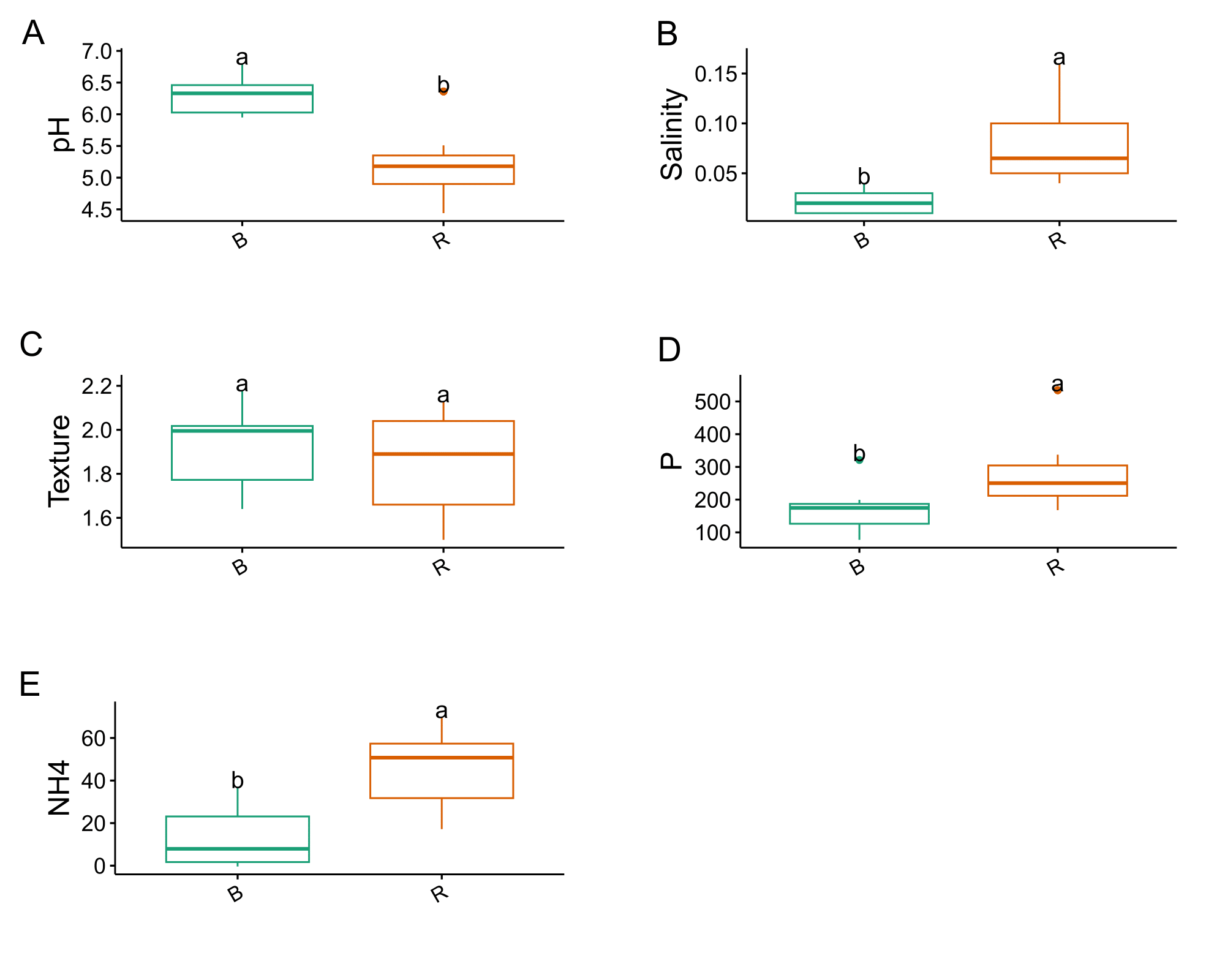


Supp. fig. 1 Box plots showing the differences in various physicochemical properties between bulk soil (B) and rhizosphere (R): Significant differences (p < 0.05) between bulk soil and rhizosphere are indicated by different lowercase letters (a, b) above the box plots. The properties include: A) pH; B) Salinity; C) Texture; D) Phosphorus (P); E) Ammonium (NH₄⁺).


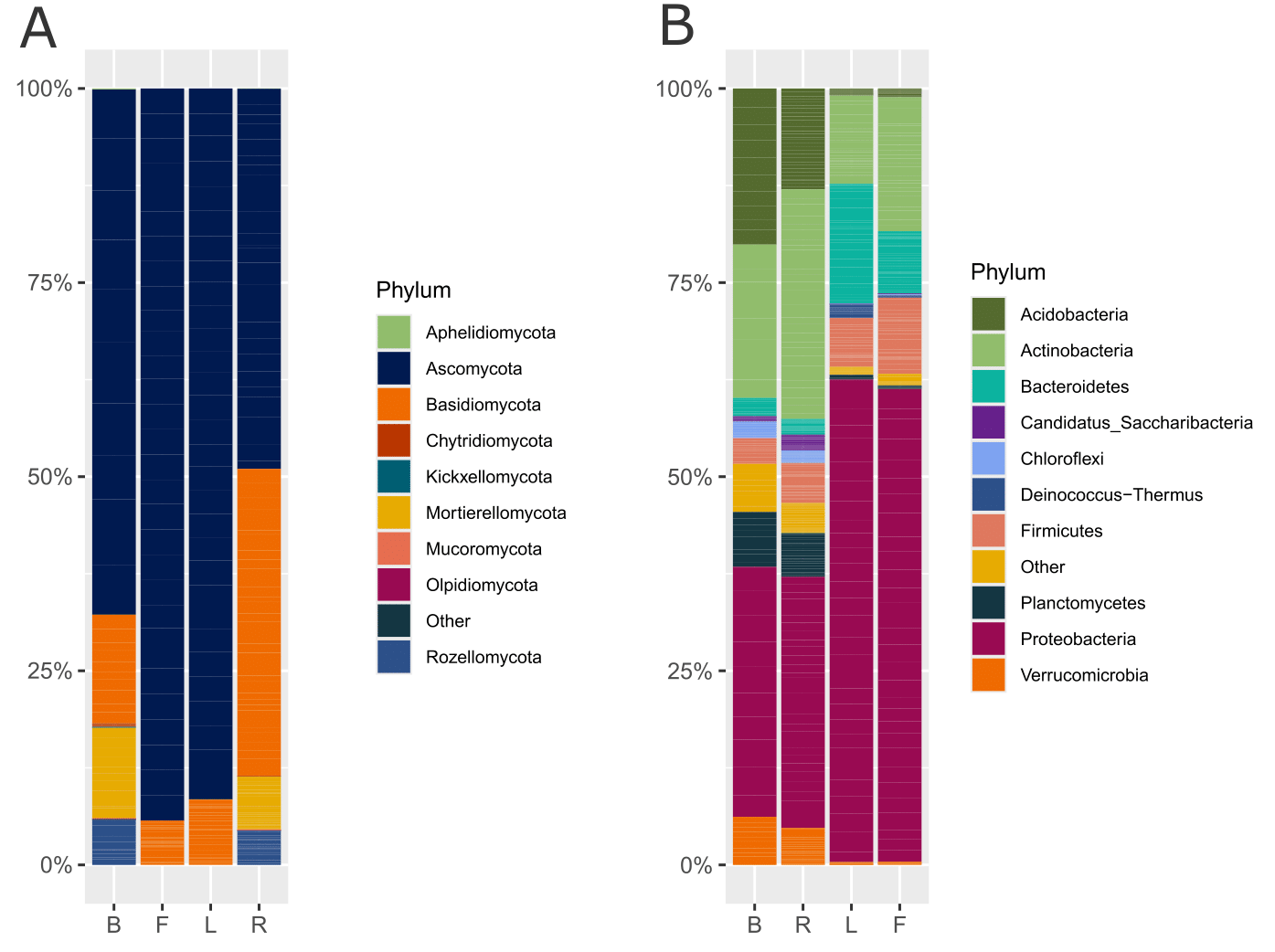


Supp. fig.2 Phylum-level composition of fungal and bacterial communities across different plant-associated compartments in blueberry cultivars (B: bulk soil; R: rhizosphere; L: leaf epiphytes; F: fruit epiphytes). Only the ten most abundant fungal phyla and the eleven most abundant bacterial phyla per compartment are shown; A) Stacked bar charts showing the relative abundance of fungal phyla across different blueberry compartments; B) Stacked bar charts showing the relative abundance of bacteria phyla across different blueberry compartments.


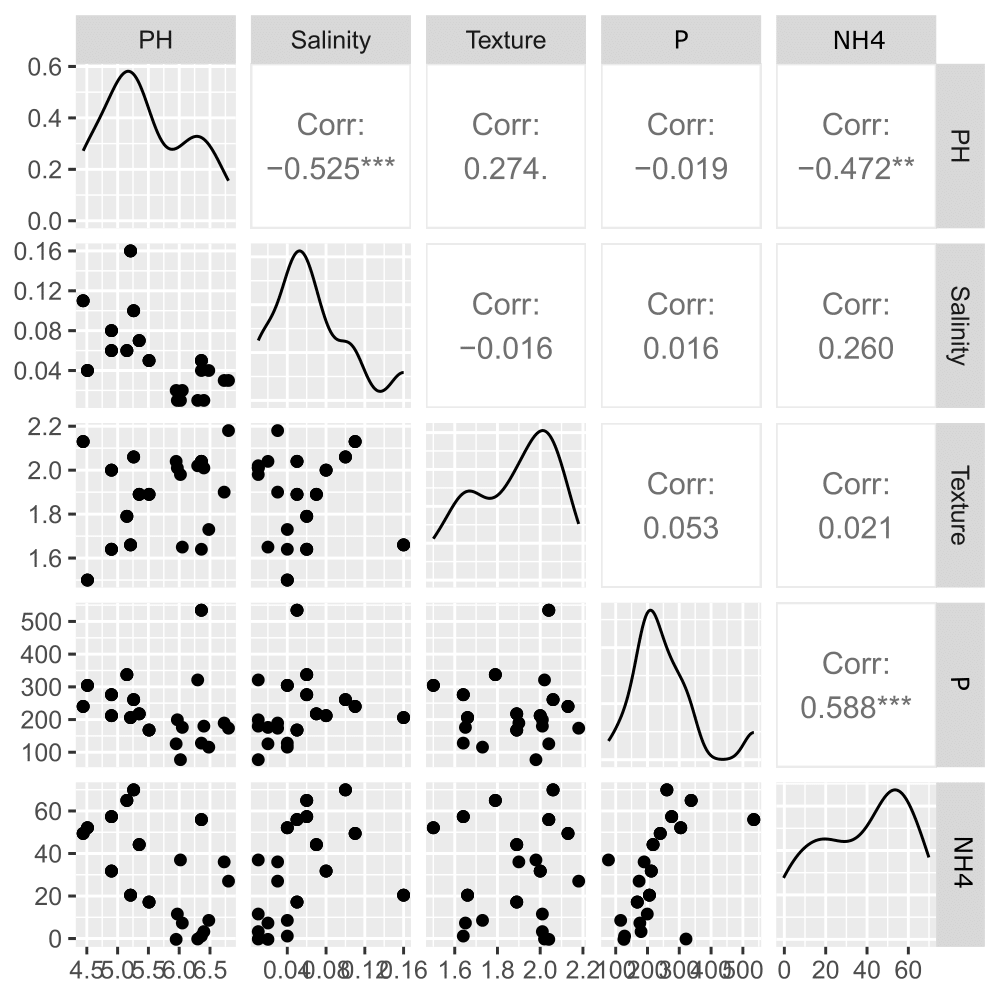

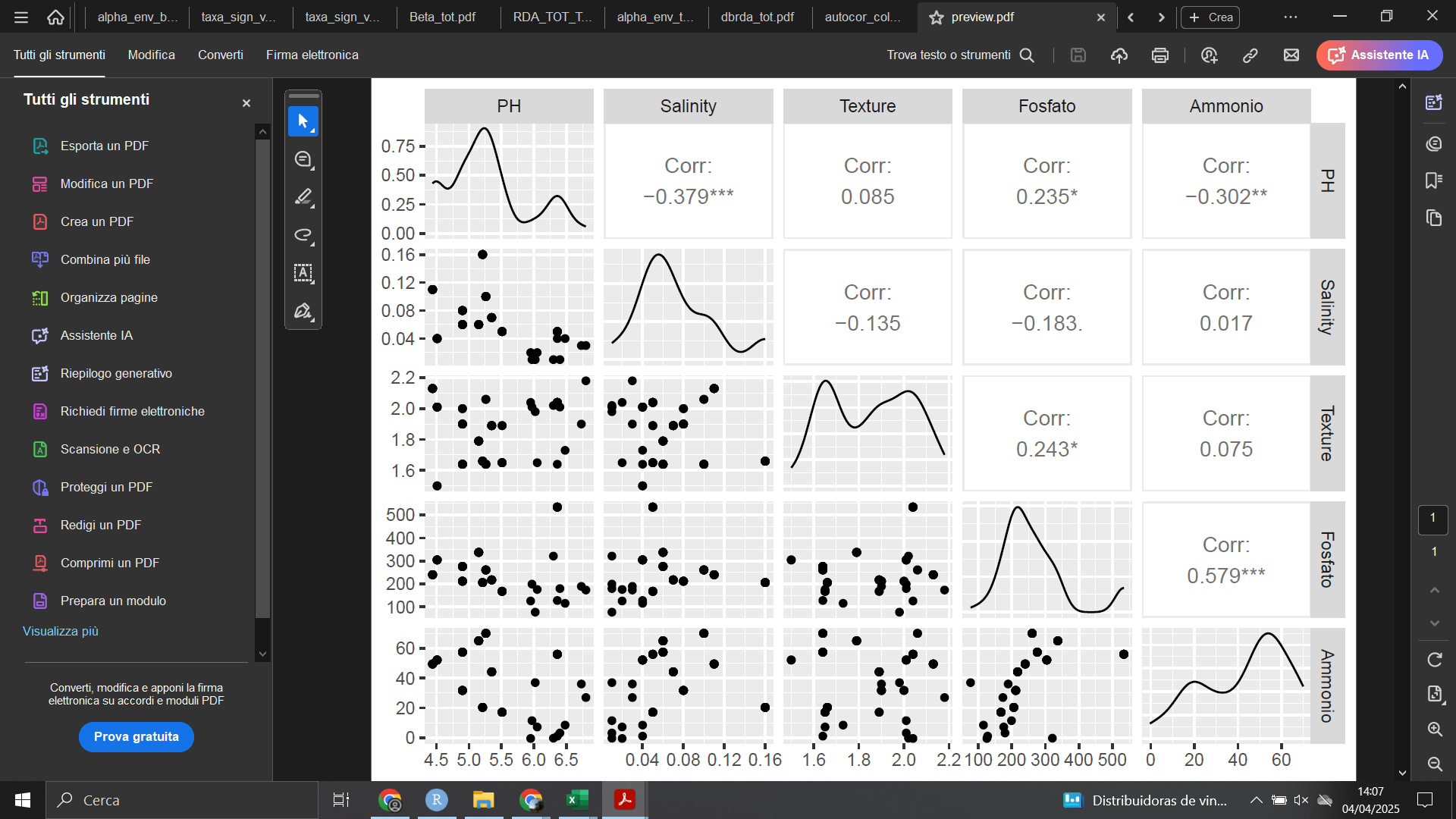


Supp. Fig. 3 Pairwise correlation matrix showing the relationships between different soil physicochemical properties: pH, salinity, texture, phosphate (P), and ammonium: The lower triangle displays scatter plots visualizing the relationships between each pair of variables, while the upper triangle shows the Pearson correlation coefficients (Corr:). The diagonal panels present density plots for each individual variable. Asterisks indicate the level of statistical significance: *** p < 0.001, ** p < 0.01, * p < 0.05.


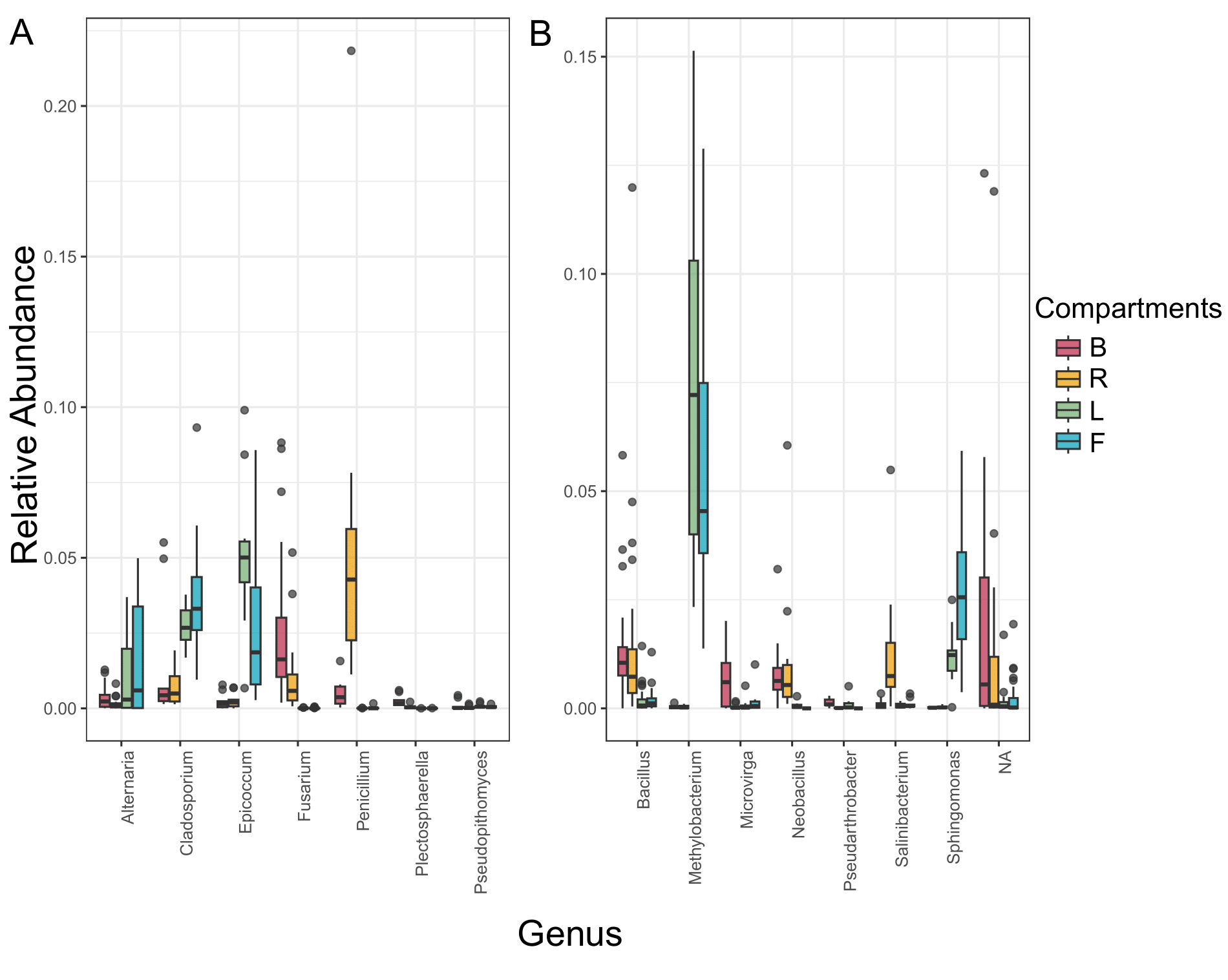


Supp. Fig. 4 Boxplots showing the relative abundance of ASVs shared across all compartments, grouped at the genus level. A) Relative abundance of fungi ASV grouped at genus level and shared between all the compartments; B) Relative abundance of bacteria ASV grouped at genus level shared between all the compartments.
